# Activation of Toll-Like Receptors and Inflammasome Complexes in the Diabetic Cardiomyopathy-Associated Inflammation

**DOI:** 10.1155/2014/847827

**Published:** 2014-03-12

**Authors:** J. Fuentes-Antrás, A. M. Ioan, J. Tuñón, J. Egido, Ó. Lorenzo

**Affiliations:** ^1^Cardiovascular Research Laboratory, IIS-Fundación Jiménez Díaz, Autónoma University, Avenida Reyes Católicos 2, 28040 Madrid, Spain; ^2^Spanish Biomedical Research Centre in Diabetes and Associated Metabolic Disorders (CIBERDEM) Network, Avenida Reyes Católicos 2, 28040 Madrid, Spain

## Abstract

Diabetic cardiomyopathy is defined as a ventricular dysfunction initiated by alterations in cardiac energy substrates in the absence of coronary artery disease and hypertension. Hyperglycemia, hyperlipidemia, and insulin resistance are major inducers of the chronic low-grade inflammatory state that characterizes the diabetic heart. Cardiac Toll-like receptors and inflammasome complexes may be key inducers for inflammation probably through NF-*κ*B activation and ROS overproduction. However, metabolic dysregulated factors such as peroxisome proliferator-activated receptors and sirtuins may serve as therapeutic targets to control this response by mitigating both Toll-like receptors and inflammasome signaling.

## 1. Introduction

Cardiac complications are the leading cause of morbidity and mortality in diabetic patients [1]. First introduced by Rubler et al. in 1971 [2], diabetic cardiomyopathy (DCM) is characterized by the direct effect of diabetes on cardiac structure and function in the absence of coronary artery disease, hypertension, or other cardiac pathologies. DCM entails the damage of the myocardium through fibrosis, steatosis, apoptosis, and hypertrophy [3] and results from the switch of substrate supply to free fatty acids (FFA) that follows the reduced levels of insulin, glucose transporters, and glucose consumption [4, 5]. Subsequent disruption of calcium homeostasis and myocardial remodeling leads to a progressive impairment of ventricular myocyte contractility that may result in heart failure [6–8]. In addition, an increasing body of evidence suggests a potential link between oxidative energy metabolism dysregulation and chronic low-grade inflammation [4, 9].

Inflammatory signaling in cardiomyocytes usually occurs as an early response to myocardial injury and entails cytosolic and mainly mitochondrial reactive oxygen species (ROS) overproduction [10, 11]. Classical following steps mainly involve increased activation of the proinflammatory nuclear transcription factor-*κ*B (NF-*κ*B), and the related expression of cytokines (i.e., tumour necrosis factor-*α* (TNF*α*), interleukins (IL-1*β*, IL-6), and chemokines (i.e., MCP-1)), adhesion molecules (i.e., selectins and adhesion molecules (ICAM-1, VCAM-1)), and successive migration of leukocytes into the myocardium [12, 13]. Migrated monocytes can further develop into tissue macrophages, which can then be polarized into two main groups, M1 and M2, accounting for their trend towards inflammation or healing, respectively. We and others have reported that myocardial inflammation develops in human patients and experimental models of type 1 (T1DM) and type 2 (T2DM) diabetes mellitus [8, 14, 15]. There is evidence that chronic progression of hypertrophy, fibrosis, and ventricular dysfunction is correlated with a local increase in cytokines [16] and activation of NF-*κ*B [17, 18]. General inflammatory stimuli in the diabetic heart include hyperglycaemia, hyperlipidemia, ROS, angiotensin II, and endothelin-1 [4, 19]. Activation of Toll-like receptors (TLRs) and the inflammasome complex has recently been proposed to be central in cardiac inflammation and likely in the pathogenesis of DCM.

## 2. Toll-Like Receptors and Cardiac Inflammation

**Figure 1:**
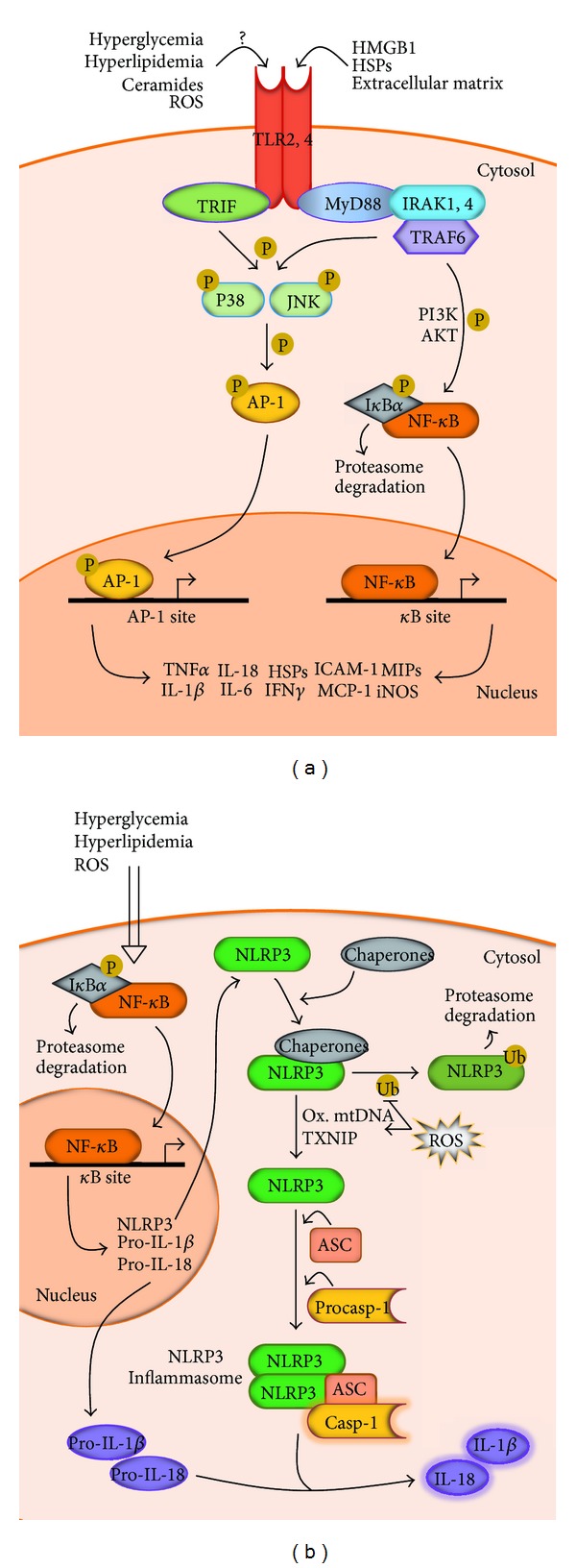
TLRs and NLRP3 inflammasome activation in the proinflammatory myocardium. (a) Activation of TLR2 and TLR4, (b) and NLRP3 inflammasome complexes in cardiomyocytes.

TLRs are membrane-anchored proteins present in several cell types ranging from macrophages and T and B cells to nonimmune cells such as cardiomyocytes [20, 21]. They work as pattern recognition receptors (PRRs) implicated in tailoring innate immune signaling [22]. TLRs elicit conserved inflammatory pathways culminating in the activation of NF-*κ*B and activating protein-1 (AP-1). TLR ligands include high-mobility group B1 (HMGB1), heat shock proteins (HSP60, HSP70), endotoxins, and extracellular matrix components [23]. Also ROS can modify membrane components and cause the release of factors that interact with and activate TLRs. In this sense, it has been shown that TLR2 participated importantly in the mechanism of ROS-induced activation of NF-*κ*B and AP-1 [24]. The proximal events of TLR-mediated intracellular signaling are initiated by interactions with cytosolic adapters, mainly myeloid differentiation primary response protein 88 (MyD88) [25]. MyD88 recruits the IL-1R-associated kinase (IRAK) and TNFR-associated factor (TRAF) to induce ubiquitination and proteasomal degradation of the inhibitors of NF-*κ*B (I*κ*Bs), thus enabling NF-*κ*B translocation into the nuclei and further ROS generation [25] (Figure 1(a)). The isoforms predominantly expressed in cardiomyocytes are TLR2 and TLR4, although up to ten cardiac TLR mRNAs have been identified in several clinical contexts including obesity and T2DM [26–29]. TLR2 and TLR4 have a central role in the pathogenesis of diverse heart disorders. Both are strongly upregulated in chronic dilated cardiomyopathy and heart failure [30], serving as upstream inducers of a large variety of proinflammatory molecules including ICAM-1, chemokines, TNF*α*, interleukins, HSPs, interferon-*γ* (IFN*γ*), and inducible nitric oxide synthase (iNOS) [21, 28, 31]. Activation of TLR2 and TLR4 eventually leads to reduction of ejection fraction through NF-*κ*B-dependent mechanisms [31, 32]. However, the specific distinction of the mechanisms and targets between TLR2 and TLR4 in cardiac inflammation is a rapidly evolving knowledge that presents some divergence. Boyd et al. reported that stimulation of TLR2 and TLR4 in HL-1 cardiomyocytes decreased contractility and initiated NF-*κ*B-dependent inflammatory response, involving upregulation of ICAM-1, chemokines, and macrophage inflammatory protein-2 (MIP-2). However, only TLR4 activation induced the proinflammatory cytokine IL-6 [31]. More recently, Ma et al. [30] uncovered the differential effects of TLR2 and TLR4 in a doxorubicin-induced mice model of chronic dilated cardiomyopathy. TLR2 blockade reduced myocardial expression of a variety of proinflammatory factors including IFN*γ* and MCP-1. Conversely, TLR4 blockade increased secretion of MCP-1, IL-13, and transforming growth factor-*β*_1_ (TGF*β*_1_). Besides structural cardiomyopathies, TLR2 and TLR4 have progressively gained credit as important contributors to entities of metabolic nature such as cardiac lipotoxicity. In this line, TLR4 knockdown abrogated NF-*κ*B-dependent inflammatory response and lowered insulin resistance in high-fat fed mice [33].

### 2.1. Activation of TLRs in DCM

Several studies have addressed the role of TLRs in cardiac inflammation using models of T1DM, T2DM, and obesity, which share an environment characterized by high circulating levels of glucose and FFA and elevated tissue levels of ceramides. Although no direct interaction between glucose and FFA with TLRs has been described [34], high levels of glucose and lipids have been shown to stimulate TLR2 and TLR4 [33, 35, 36], thus suggesting the existence of unknown intermediates. High-fat diet-induced obese mice exhibited myocardial macrophage infiltration as well as higher expression levels of TLR4, MyD88, and IL-6 [37]. Consistent with this, both diabetic TLR2 and TLR4-deficient mouse hearts showed lower triglyceride accumulation during the early stages of diabetes, as well as restricted leukocyte infiltration and a marked decrease of NF-*κ*B and MyD88 and phosphorylation of IRAK1 [20, 38]. Different studies in T1DM mice models show that TLR4 silencing prevents cardiac lipid accumulation, hyperglycemia-induced myocardial apoptosis, and ventricular remodeling and dysfunction. It also suppresses the diabetic upregulation of NADPH oxidase activity and thus ROS production [20, 39]. Furthermore, genetic analysis of patients has pointed an association between TLR4 polymorphisms, diabetes prevalence, and the severity of chronic cardiomyopathy [40, 41]. However, besides the focus on the alterations of cardiomyocytes, leukocyte activation and transmigration into the diabetic myocardium constitute a pivotal process in the inflammatory response. Hyperglycemia has been shown to upregulate TLR2, TLR4, MyD88, and IRAK-1 phosphorylation and TLR-mediated transactivation of NF-*κ*B in human monocytes from T2DM patients [28, 42]. Concurrently, TLR2 increased in mononuclear cells from long-standing T1DM patients [43]. In macrophages from a model of nonobese T2DM mice, Mohammad et al. described a ten-fold increase of TLR4 and higher levels of cytokines, while anti-inflammatory IL-10 was downregulated [21]. Consistent with this view, monocytes from T2DM patients also exhibited significant increment in proinflammatory cytokines and TLR2 and TLR4 ligands (HMGB1 and HSPs) [28]. Similar to what occurred in cardiomyocytes, siRNA knockdown of TLR2 and TLR4 led to decreased NF-*κ*B activity and IL-1*β* release in monocytes [42]. Therefore, it seems that TLRs may be activated in both cardiomyocytes and leukocytes, in DCM-associated cardiac inflammation ten-fold.

## 3. Inflammasomes and Cardiac Inflammation 

The inflammasome is a group of multimeric protein complexes composed of a cytoplasmic receptor of the Nod-Like Receptor (NLR) family, an adaptor protein termed ASC (Apoptosis-associated Speck-like protein containing an N-terminal caspase recruitment domain CARD), and procaspase-1 [44]. The best characterized complex is the NLRP3 inflammasome, which has been identified in a wide range of cells including macrophages, cardiofibroblasts, and cardiomyocytes [45–48]. NLRP3 has been reported to be held in an inactive state by cytoplasmic chaperones. Once NLRP3 is freed, subsequent oligomerization leads to the recruitment of procaspase-1, thus promoting autocleavage and activation [44]. Active caspase-1 can eventually process IL-1*β* and IL-18 precursors, serving as enhancer of multiple proinflammatory pathways including NF-*κ*B, mitogen-activated protein kinase (MAPK), IFN*γ*, chemokines, and ROS and also promoting insulin resistance [49] (Figure 1(b)). NLRP3 can be activated by long-chain saturated FA (i.e., palmitate), ceramides, modified LDL, and hyperglycemia [50–52]. However, NLRP3 does not have a known direct ligand and it requires two-checkpoint activation process including a priming step and a second activation step [53]. NF-*κ*B is the traditional priming signal for the transcription of the NLRP3 gene [54], whereas novel mechanisms have recently emerged as a second step. These are based on posttranslational activation of NLRP3 by deubiquitination [55], oxidized mitochondrial DNA [56], and potential ligands such us thioredoxin-interacting protein (TXNIP) [57]. In addition, a recent study by Bauernfeind et al. has revealed that NLRP3 expression is critically regulated by myeloid specific microRNA-223 [58]. Nevertheless, to date, most data about NLRP3-inflammasome implication in heart disease and inflammation come from murine models of ischemic damage and dilated cardiomyopathy [48, 59, 60]. In a model of dilated cardiomyopathy, NLRP3 ablation was related to a general reduction in proinflammatory cytokines maturation, reduced mononuclear infiltrate, maintained myocyte organization and structure, and preserved systolic performance [48]. In addition, these hearts increased phosphorylation of I*κ*B*α*, what is consistent with NF-*κ*B regulated NLRP3 gene expression. Further evidence shows upregulation of the NLRP3-inflammasome effector caspase-1 in murine and human myocardial infarction [61]. In this study, deletion of endogenous caspase-1 consistently ameliorated the ventricular function of the postinfarcted heart. However, many aspects are in need of further clarification. NLRP3 mRNA levels have been found to be markedly diminished in heart samples from the right atrium of patients undergoing coronary bypass surgery [60]. Moreover, Zuurbier et al. have recently reported that deletion of NLRP3 resulted in decreased myocardial IL-18 and IL-6 levels, but this effect was not observed for IL-1*β* and TNF*α* levels. Also, deletion of the ASC component did not downregulate IL-6, IL-1*β*, or TNF*α* [59]. Despite this, gene polymorphisms and mutations in the human NLRP3-inflammasome have been shown to be associated with an increase of IL-1*β* and IL-18, higher levels of C-reactive protein (CRP), and severe inflammation [62–64].

### 3.1. Activation of NLRP3-Inflammasomes in DCM

Not much research has been done to assess the plausible implication of inflammasomes in experimental models of DCM. However, as for TLRs, several recent studies have emphasized that NLRP3 inflammasomes might represent the link between inflammation and metabolic disorders such in the diabetic heart. It is known that NLRP3 signaling affects glycolysis and insulin sensitivity and simultaneously enhances both local myocardial cytokine levels and infiltration by macrophages [51, 65]. Recent data also suggest that NLRP3 is responsible for sensing obesity-associated host-derived inducers of caspase-1, such as ROS and lipotoxic ceramides and palmitate [66]. In fact, NLRP3 inflammasomes have been proposed to sense and mediate downstream inflammatory events of glycotoxicity and lipotoxicity during the pathogenesis of T2DM [45, 57]. Cardiac NLRP3, caspase-1, and IL-1*β* expression was substantially increased in obese mice and human subjects [45]. Moreover, caloric restriction and exercise-mediated weight loss in obese individuals with T2DM were shown to effectively reduce the expression levels of NLRP3 [67]. In contrast to the scarce contributions in cardiomyocytes, research on NLRP3 inflammasomes has intensively focused on inflammatory cells. NLRP3 has been reported to increase effector T-cell number, thus eliciting macrophage transmigration. Further, NLRP3 upregulates the pool of proinflammatory cytokines such as IL-1*β*, IL-18, and IFN*γ* and promotes insulin resistance in M1 macrophages [45]. In addition, both ceramides and palmitate require an intact NLRP3 signaling to induce caspase-1 activation and IL-1*β* and IL-18 release from macrophages [45, 66]. Thus, NLRP3 inflammasome may also participate in the cardiomyocyte and monocyte response in DCM-associated inflammation.

## 4. Potential Crosstalk between TLRs, Inflammasomes, and Metabolic Dysregulation in DCM

**Figure 2:**
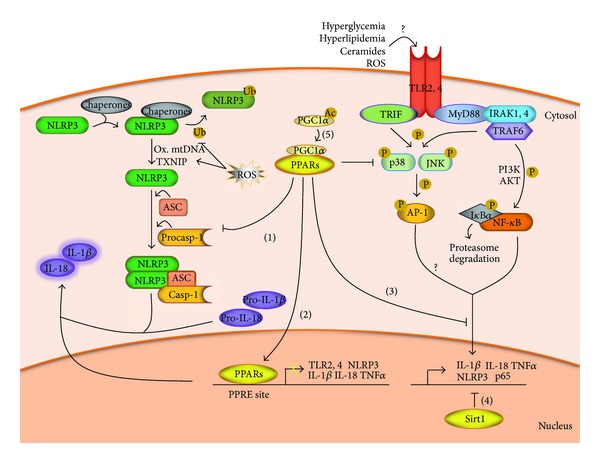
Crosstalk between TLRs, NLRP3 inflammasomes and dysregulated metabolic factors in DCM. PPARs and Sirt1 may control NLRP3 inflammasome and TLR pathways by interfering with the inflammasome assembly (1), proinflammatory gene overexpression (2), and NF-*κ*B signaling (3-4). In addition, Sirt1 could mediate PPARs activation by PGC1*α* deacetylation (5).

Interestingly, TLR2 and TLR4-mediated ROS generation and NF-*κ*B transactivation upregulate NLRP3 pathway through multiple direct and indirect mechanisms, which account for both NLRP3 priming and the secondary steps of activation (Figure 2). First, ROS/NF-*κ*B has been reported to enhance the expression of NLRP3 and caspase-1 target pro-IL-1*β*, and NF-*κ*B sites in* NLRP3* promoter have been identified [36, 54, 67]. Second, ROS/NF-*κ*B facilitates NLR posttranslational deubiquitination [55]. And third, ROS/NF-*κ*B increases the amount of TXNIP and oxidized mitochondrial DNA, which might serve as ligands of NLRP3 [56, 57]. Thus, NLRP inflammasome activation is likely to be a key outcome of TLR stimulation in DCM. Moreover, another connection between TLRs and inflammasomes may be through metabolic dysregulated factors, such as peroxisome proliferator-activated receptors (PPARs) and sirtuins (Sirts) (Figure 2). Activation of PPARs is a key process in the myocardial switch of substrates in DCM and has recently emerged as a link between metabolism disturbance and pathological inflammatory/oxidative phenomena [68]. The PPAR transcription factor family is extensively known to regulate cardiac metabolism, mainly through PPAR*α* and PPAR*β*/*δ* isoforms together with PGC-1*α* coactivator [69]. PPAR*α*/PGC-1*α* leads to transcriptional induction of pyruvate dehydrogenase kinase-4 (PDK4), FAT/CD36 transporter, and FFA oxidation enzymes [70], thereby facilitating mitochondrial FFA import and *β*-oxidation-dependent metabolism in expenses of glucose assimilation. Thus, NF-*κ*B and p38-mediated PPAR*α*/PGC-1*α* inhibition has been described as an important pathological mechanism in DCM progression [71]. In addition, a wide body of evidence indicates that PPARs mitigate inflammation. PPARs lower nuclear factor of activated T-cells (NFAT) signaling and prevent the expression of NADPH oxidase subunits, resulting in ROS amelioration [72, 73]. PPARs also downregulate TLR2 and TLR4 signaling by either blocking TLR expression or its NF-*κ*B and AP-1-dependent pathways [74–76]. The effects on NF-*κ*B seem to be mediated through direct physical interactions, sequestration of NF-*κ*B coactivators, and transcriptional control of NF-*κ*B-related proinflammatory genes [77–79]. Other DNA-independent mechanisms to inhibit NF-*κ*B include activation of ERK-MAPK pathway, mainly by impairing phosphorylation of factors such as p38- and JNK-MAPK [4]. Notably, these are also targets of TLR-Myd88/IRAK signaling in the heart [18, 80]. Further direct evidence from obese T2DM mice has demonstrated that PPAR*β*/*δ* and PPAR*γ* downregulate both TLR2 and TLR4 signaling [75, 81]. Moreover, TNF*α* and IL-1*β* have been reported to be clearly decreased upon activation of PPAR*α* [82, 83], which can be linked with impaired NF-*κ*B-dependent induction of NLRP3 inflammasome. In a model of chronic high-fructose-induced diabetic mice, Collino et al. described that PPAR*β*/*δ* stimulation attenuated NLRP3-dependent caspase-1 activation and IL-1*β* production [74]. Also, the NLR family promoter harbours binding sites for PPAR*γ* [84]. More complex evidence regards the interference of the inflammasome assembly by phospholipase C, cyclic AMP, and protein kinase C, which are known regulators and targets of PPARs [85–87].

In addition to PPARs, Sirts may constitute another alleged nodal connection between metabolism and TLR and/or inflammasome-dependent inflammation [88] (Figure 2). Moreover, Sirts have been largely reported to interfere with the molecular pathogenic substrate of heart failure and thereby ameliorate cardiac outcome [89]. Epigenetic modulation by this class III deacetylase limits oxidative stress and inflammatory responses by targeting a relevant set of transcription factors including NF-*κ*B, PPARs, and PGC-1*α* [90]. Sirtuin-1 (Sirt1), the most studied Sirt in the heart, works as an energy sensor and supports oxidative energy metabolism through PPAR*α*/PGC-1*α* and AMP-activated protein kinase (AMPK) signaling, which also contribute to inhibit NF-*κ*B and inflammation [91]. Mice overexpressing Sirt1 and exposed to high-fat diet show attenuated lipid-induced inflammatory responses [92]. Also, Sirt1 was reported to stimulate antioxidants manganese superoxide dismutase (MnSOD) and nuclear respiratory factor-1 (Nrf1) in the heart, downregulating NF-*κ*B targets TNF*α* and IL-6 [93]. Several mechanisms for the modulation of NF-*κ*B signaling by Sirt1 have been described. First, Sirt1 has been associated with PPAR*α*-dependent inhibition of p65 subunit of NF-*κ*B [94]. Second, Sirt1 may directly modulate NF-*κ*B-dependent immune responses and coupled ROS production by deacetylating p65 [95]. And third, Sirt1 can negatively regulate the expression of TNF*α* and IL-1*β* by binding to specific sites in their promoters [96]. Further connections between Sirt1, TLRs and inflammasomes include Sirt1 downregulation by palmitate-induced miR-195 and Sirt1 cleavage by caspase-1 [92, 97]. Taken together, activation of both PPARs and Sirt1 may control the TLR and inflammasome-dependent pathways of inflammation in DCM, which may be useful for a therapeutic target.

## 5. Prospective Therapeutic Targets for DCM

Despite the prolific area of research linking inflammation, diabetes and metabolic heart disease, the drugs currently employed in the care of diabetic patients have not generally been based on an anti-inflammatory strategy. Pharmacological modulation of TLRs undoubtedly arises as a highly attractive therapeutic strategy for DCM. In this regard, several TLR antagonists have been assessed in diverse models of myocardial contractile dysfunction. Selective inhibition of TLR2 by immunoglobulin G (IgG) has been successfully attempted to ameliorate NF-*κ*B and leukocyte infiltration in ischemic murine hearts [98]. TLR4 antagonists, eritoran, and geldanamycin resulted in attenuated myocardial inflammatory responses including reduced p-JNK and NF-*κ*B nuclear translocation and decreased gene transcripts of TNF*α*, IL-1*β*, IL-6, MCP-1, MIP-1*α*, and MIP-2 [99, 100]. These data underscore the potential benefit of blocking TLR signaling for DCM. However, an increasing number of TLR inhibitors are not being proportionally tracked by studies measuring their impact in animal models of cardiac disease [101, 102]. The discovery of novel mechanisms for common drugs also paves the way for potential therapeutic strategies. For example, statins attenuated the upregulation of TLR4 and TLR2, inhibited NF-*κ*B, and decreased the circulating levels of TNF*α*, MCP-1, and CRP in a mice model of dilated cardiomyopathy [103, 104]. Also, angiotensin II receptor blocker valsartan decreased TLR4-mediated NF-*κ*B activity and subsequent cytokine release in a rat model of ischemic heart [105]. In addition, cumulative evidence on NF-*κ*B and TNF*α* targeting suggests the therapeutic value of specific modulation of TLR downstream effectors. Triptolide, a potent NF-*κ*B immunomodulator and TNF*α* monoclonal antibody treatment significantly decreased TNF*α*, IL-1*β*, ICAM-1, VCAM-1, and subsequent myocardial infiltration by macrophages and T-cells in diabetic hearts [106, 107].

No NLR antagonist has been identified yet, and increasing efforts are being invested as a result of successful blockade of downstream effectors IL-1*β* and caspase-1 in DCM. Very recent evidence reported that intravenous IgG therapy protected neurons in an experimental model of stroke through a mechanism involving suppression of the NLRP3 inflammasome activity [108]. Moreover, the anti-IL-1*β* Anakinra [109] and Gevokizumab [110] clinical trials resulted in reduced TNF*α*, IL-6, IL-1*β*, and CRP. However, despite reducing biomarkers of heart disease, they did not restore hyperglycemia. Nonimmune antagonists of the inflammasome machinery are equally compelling. Pralnacasan, a caspase-1 blocker, has been reported to attenuate inflammation in a model of DCM by reducing IL-1*β*, IL-18, TNF*α*, and IFN*γ* levels, intracardiac macrophage, and lymphocyte infiltrates and also to improve insulin sensitivity [107, 111]. Novel anti-inflammasome properties have been described for classical antidiabetics such as metformin and sulfonylurea. Metformin may affect NLRP3 signaling by enhancing autophagy through AMPK [66, 112] or increasing Sirt1 action [113, 114]. In this line, AICAR, an AMPK agonist, could also restore the formation of autophagosomes and thereby inhibit both caspase-1 and ROS generation in palmitate-treated macrophages [66]. Sulfonylurea glyburide also suppressed the NLRP3-dependent caspase-1 activation and IL-1*β* release [115]. Finally, Jourdan et al. showed that the blockade of cannabinoid receptor type 1 (CB1R) lowered the levels of NLRP3, ASC, IL-1*β*, IL-18, NF-*κ*B, and caspase-1 in macrophages from ZDF rats [116].

Beyond the regulation of TLRs and inflammasomes, therapeutic benefit of PPARs and Sirt1 stimulation on T2DM and its cardiac complications has been reported in recent years [117–120]. Interestingly, a PPAR*α* agonist, fenofibrate, decreased TLR4 and MyD88 expression in a model of multiple sclerosis [121]. PPAR*γ* agonist pioglitazone substantially inhibited the expression of TLR2, TLR4, MyD88, and NF-*κ*B in macrophages from obese T2DM mice [75]. Moreover, PPAR*β*/*δ* agonist GW0742 impaired NLRP3 inflammasome activity in high-fructose diet-induced diabetic mice [74]. Since TLR4 downregulation was identified as an anti-inflammatory mechanism of the insulin-sensitizer incretin glucagon-like peptide-1 (GLP-1) [122], and considering that a PPAR*β*/*δ* agonist markedly upregulated GLP-1 in obese T2DM mice [123], it is possible that PPAR*β*/*δ* stimulation may be a valid therapeutic tool for DCM. In the same way, a bulk of emerging evidence has identified Sirts as a future therapeutic target for diabetic complications. In this sense, small molecule activators of Sirt1 are currently being developed. ZDF rats undergoing this treatment effectively improved whole-body glucose homeostasis and insulin resistance [124]. However, no attempt to measure the impact of Sirt1 enhancement in DCM has been made.

## 6. Conclusions

TLRs and the inflammasome signaling platforms could be two main breakthroughs on cardiac inflammation. Emerging evidence supports a model in which hyperglycemia and FFA stimulate TLRs as upstream inducers of proinflammatory mechanisms in DCM. TLR-dependent NF-*κ*B and ROS appear to regulate both the priming and posttranslational steps required for the assembly and activation of the inflammasome. However, metabolic dysregulated factors such as PPARs and Sirt1 can downmodulate DCM inflammation by interfering with TLRs and inflammasome signaling. Thus, a new set of potential therapeutic approaches for DCM may include the stimulation of PPARs and Sirt1 and the inhibition of TLR2, TLR4, and NLRP3. Further, targeting proximal TLR mediators Myd88 and IRAK and the activation steps of the inflammasome may yield some clinical benefit in DCM.
